# A Case of Acute Angle Closure Secondary to Pupillary Block Caused by a Dislocated Intraocular Lens-Capsular Tension Ring Complex

**DOI:** 10.7759/cureus.72963

**Published:** 2024-11-04

**Authors:** Kana Murakami, Kazunobu Sugihara, Ayaka Shimada, Mizuki Iida, Masaki Tanito

**Affiliations:** 1 Department of Ophthalmology, Shimane University Faculty of Medicine, Izumo, JPN

**Keywords:** acute angle closure, anterior-segment optical coherence tomography (as-oct), capsular tension ring, cataract surgery, glaucoma surgery, pupillary block

## Abstract

We report a case of acute angle closure secondary to pupillary block caused by a dislocated intraocular lens (IOL)-capsular tension ring (CTR) complex. A 68-year-old woman, who had undergone cataract surgery 28 months earlier, presented with acute right eye pain and blurred vision. Examination revealed elevated intraocular pressure (IOP) of 80 mmHg, corneal edema, and anterior chamber shallowing, with anterior displacement of the IOL-CTR complex observed on anterior-segment optical coherence tomography. An anterior chamber paracentesis and subsequent vitrectomy were performed to remove the dislocated complex. After scleral fixation of a new IOL and management of an epiretinal membrane, the patient’s vision improved, with her best-corrected visual acuity reaching 1.2 and IOP stabilizing at 18 mmHg without medication. This case highlights the potential for IOL-CTR dislocation to cause pupillary block and acute angle closure, emphasizing the need for timely diagnosis and comprehensive surgical intervention to preserve visual function.

## Introduction

A capsular tension ring (CTR) is used to stabilize the capsular bag, ensuring the safety of cataract surgery and postoperative stability of the intraocular lens (IOL). It is utilized in the surgical management of conditions such as zonular dialysis due to pseudoexfoliation syndrome [1], trauma [2,3], retinitis pigmentosa [4,5], lens subluxation from various causes [6], and microspherophakia [7]. Zonulopathy has been reported in 7.3% of cases of primary angle-closure disease [8]. Therefore, CTR is also employed during cataract surgery in both primary and secondary angle-closure cases [4,5,9,10].

In some cases, the IOL-CTR complex may dislocate into the vitreous cavity during the postoperative period [11-13]. More rarely, there have been reports of intermittent or acute angle closure caused by the IOL-CTR complex [14,15]. We encountered a case of acute angle closure due to an IOL-CTR complex. In this case, pupillary block caused by iris compression from the dislocated IOL-CTR complex was clearly observed.

## Case presentation

A 68-year-old Japanese woman visited an ophthalmology clinic due to decreased vision that had persisted for two years. She was referred to Shimane University Hospital for treatment of cataracts and a shallow anterior chamber in her right eye. Her medical history included hypertension, hypercholesterolemia, hyperuricemia, and nephrectomy as a donor for kidney transplantation. She had no previous history of ocular disease.

**Figure 1:**
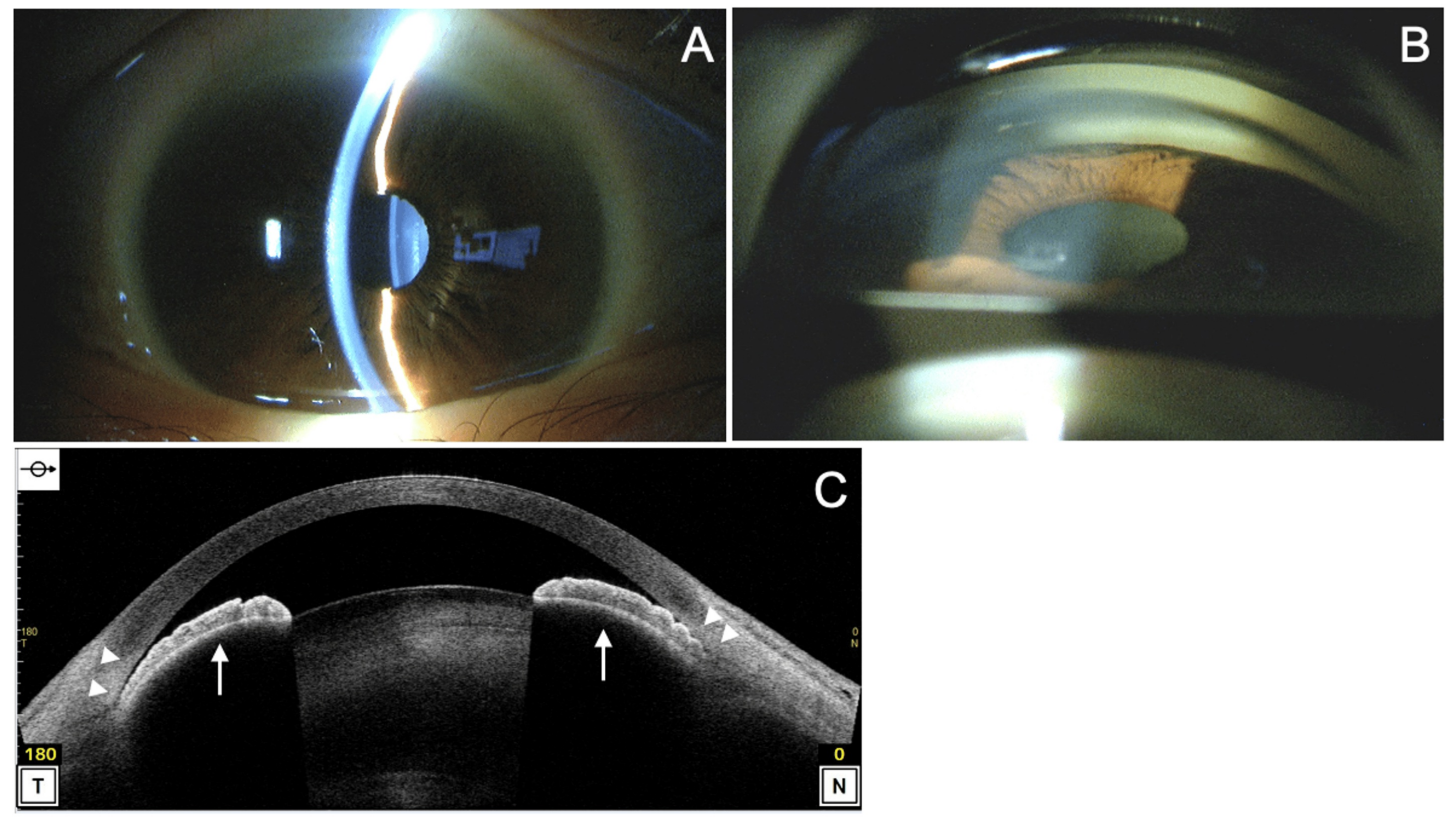
Preoperative slit-lamp (A), gonioscopy (B), and AS-OCT (C) findings of the right eye before cataract surgery. (A) Slit-lamp examination reveals cataract and shallow anterior chamber depth. IOP is 15 mmHg. (B) Gonioscopy shows a narrow angle with Shaffer grade 0. No peripheral anterior synechiae are observed. (C) AS-OCT (horizontal scan) demonstrates anterior bowing of the iris (arrows) and irido-trabecular contact (arrowheads). AS-OCT: anterior-segment optical coherence tomography; IOP: intraocular pressure

At the initial visit to our hospital, her best-corrected visual acuity (BCVA) was (0.6 × S-6.0D = C-3.0D Ax 90°) in the right eye and (1.2 × S-2.0D = C-2.0A Ax 100°) in the left eye. Intraocular pressure (IOP) measured by the Goldmann applanation tonometer was 15 mmHg in both eyes. Slit-lamp examination revealed clear corneas in both eyes, with a shallow anterior chamber in the right eye (Figure 1A) and a deep anterior chamber in the left eye. No pseudoexfoliation material was observed in either eye. Both eyes had cortical lens opacity, but no significant phacodonesis was noted. Gonioscopy showed a Shaffer grade of 0 in the right eye (Figure 1B) and grade 3 in the left eye, with no peripheral anterior synechiae (PAS) detected on compression gonioscopy. The vertical cup-to-disc ratio was 0.7 in both eyes. Axial length (OA-2000, Tomey Corporation, Nagoya, Japan) was 24.39 mm in the right eye and 24.09 mm in the left eye. Anterior chamber depth (OA-2000) was 2.54 mm in the right eye and 2.99 mm in the left eye. Corneal endothelial cell density (EM-3000, Tomey Corporation) was 2,712 cells/mm² in the right eye and 2,604 cells/mm² in the left eye, with central corneal thickness (EM-3000) measuring 533 µm in the right eye and 514 µm in the left eye. Anterior-segment optical coherence tomography (AS-OCT) (CASIA2 Advance, Tomey Corporation) revealed irido-trabecular contact and anterior bowing of the iris in the right eye (Figure 1C).

**Figure 2:**
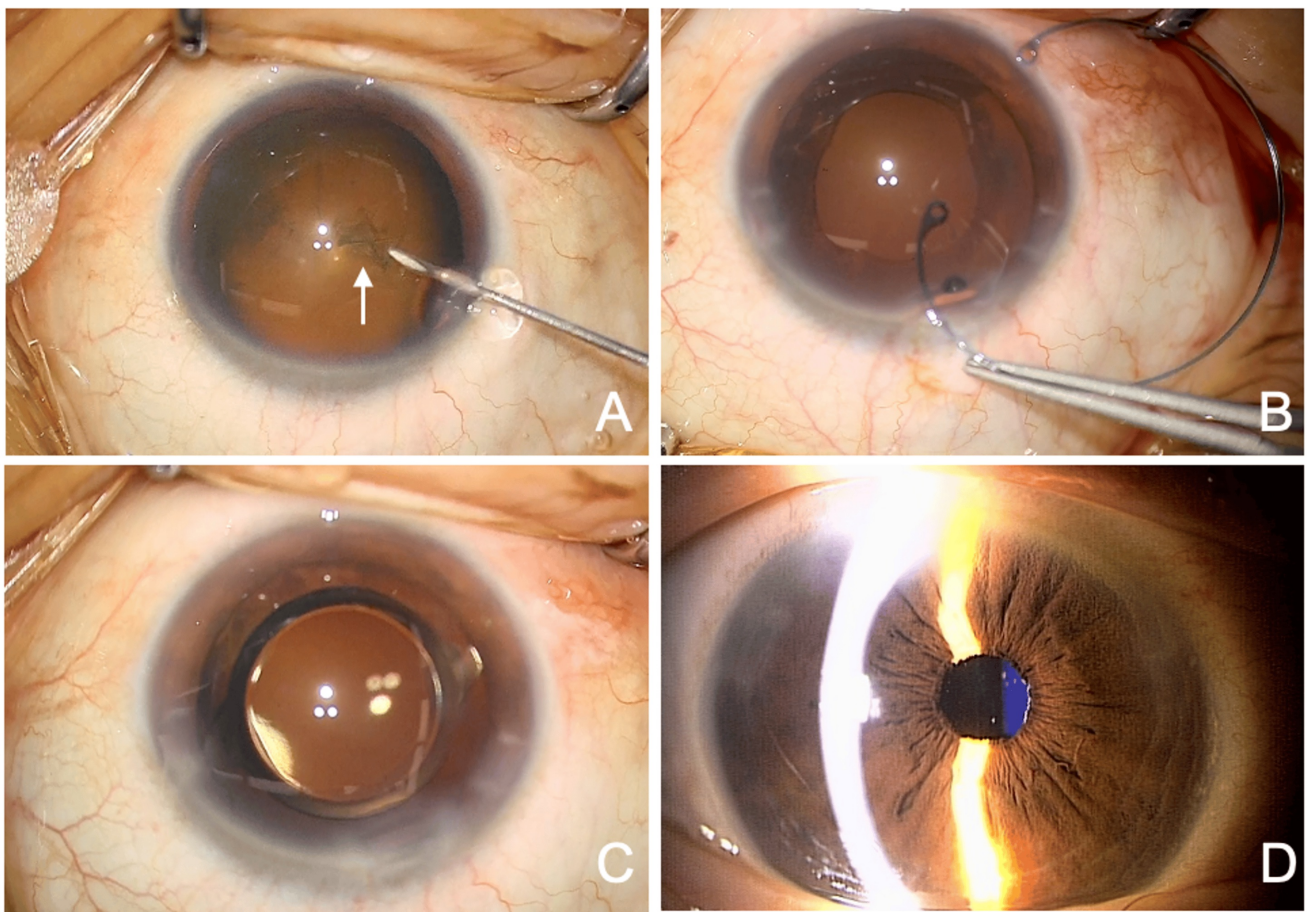
Intraoperative findings from the initial cataract surgery (right eye). (A) A continuous curvilinear capsulorhexis flap (arrow) is created using a cystotome due to zonular weakness. (B) A CTR is inserted into the capsular bag for stabilization of the capsular bag and the IOL position. (C) The IOL is well-centered and fixed in the capsular bag. (D) One week post-surgery, the anterior chamber depth is deep, and the IOP is 15 mmHg. CTR: capsular tension ring; IOL: intraocular lens; IOP: intraocular pressure

The patient was diagnosed with angle closure due to zonular weakness of unknown etiology, and cataract surgery was performed on the right eye to prevent acute glaucoma (Figures 2A-2C, Video 1). Surgery was performed using a 2.2 mm corneal incision and ultrasound phacoemulsification (Figure 2A). Due to the zonular weakness, a CTR (CTR130AD, Hoya, Tokyo, Japan) was inserted into the capsular bag to maintain the stability of the capsular bag and IOL centering (Figure 2B). A +18.5D one-piece IOL (DCB00V, Johnson & Johnson Vision, Tokyo, Japan) was implanted in the capsular bag (Figure 2C). Postoperatively, levofloxacin, betamethasone, and nepafenac eye drops were prescribed three times daily for about three weeks. One week postoperatively, the anterior chamber depth had deepened, and the IOL was well-centered (Figure 2D). The BCVA in the right eye was 1.2, and the IOP was 15 mmHg. One month after surgery, the patient was doing well and was referred back to her original ophthalmologist for follow-up care, ending her treatment at our hospital.

**Video 1:** Intraoperative findings from the initial cataract surgery (right eye). The video is being played at double speed.

**Figure 3:**
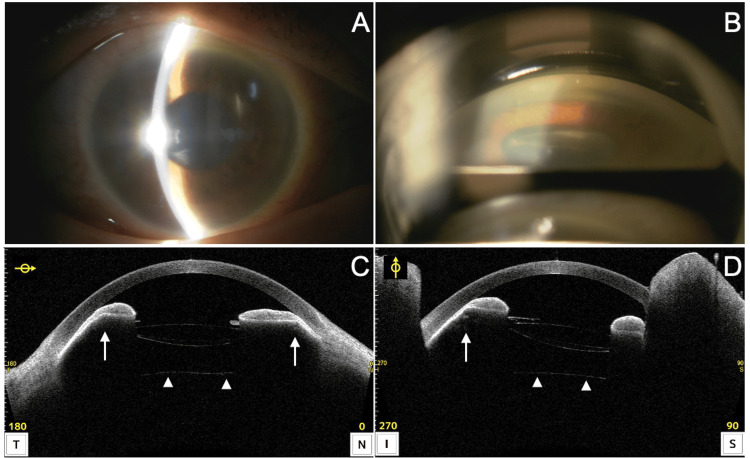
Findings during the emergency visit 28 months after cataract surgery (right eye). (A) Slit-lamp examination reveals corneal edema and a shallow anterior chamber. The IOP is 80 mmHg. (B) Gonioscopy shows that the angle structure is not visible. (C, D) AS-OCT (horizontal and vertical scans) shows anterior displacement of the IOL-CTR complex and iris compression consistent with the CTR diameter (arrows). There is no anterior displacement of the anterior vitreous membrane (arrowheads). AS-OCT: anterior-segment optical coherence tomography; CTR: capsular tension ring; IOL: intraocular lens; IOP: intraocular pressure

Twenty-eight months after the initial surgery, the patient presented to the emergency department of Shimane University Hospital at night complaining of right eye pain and blurriness that had started earlier in the evening. An ophthalmologist was consulted, and a slit-lamp examination revealed corneal edema, moderate mydriasis, and angle closure in the right eye (Figures 3A, 3B). The IOP in the right eye was 80 mmHg. AS-OCT showed evidence of anterior displacement of the entire IOL-CTR complex (Figures 3C, 3D), consistent with pupil block caused by the dislocated IOL-CTR complex (arrows). There was no anterior movement of the anterior vitreous membrane (arrowhead).

**Figure 4:**
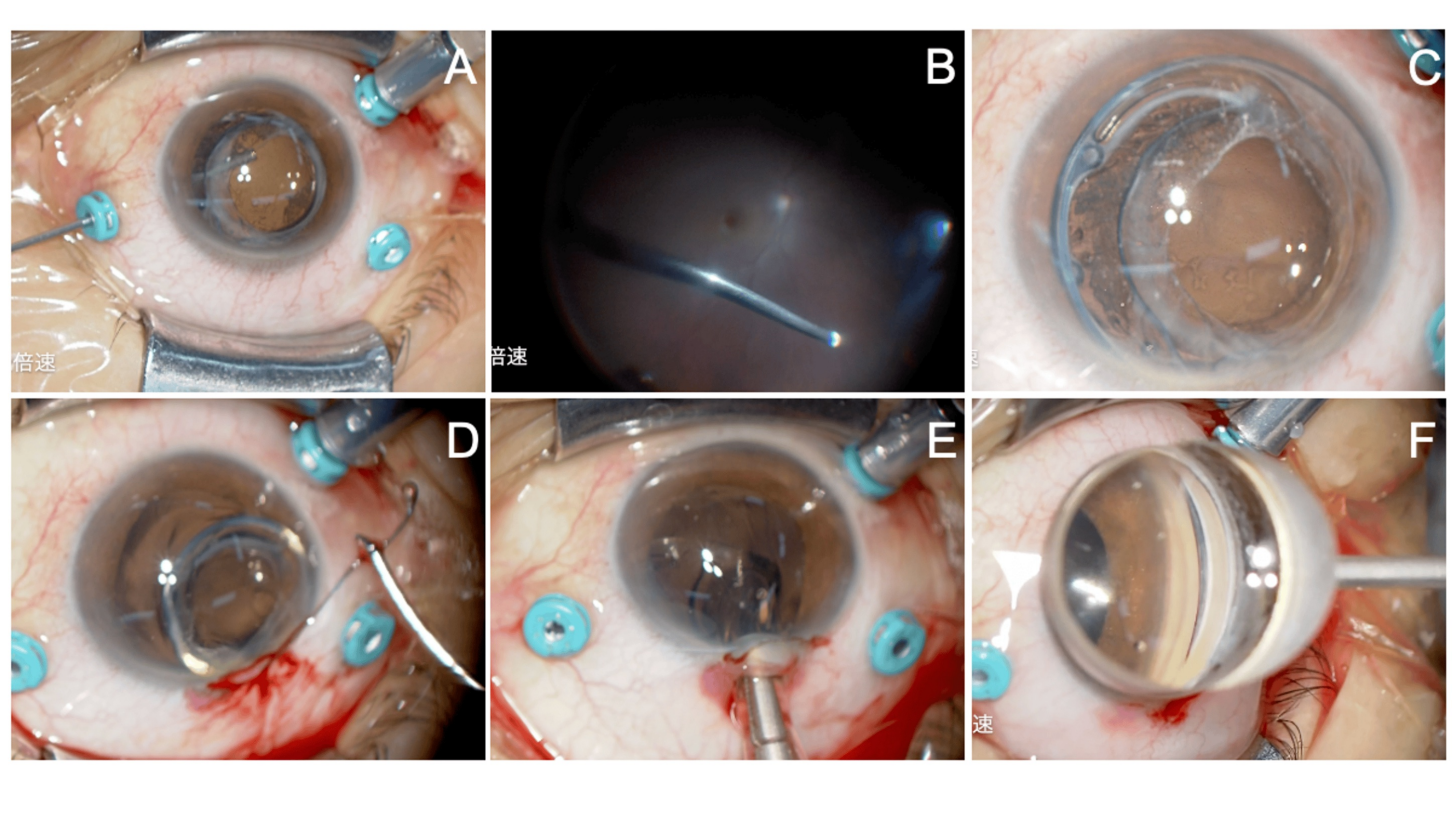
Removal of the IOL-CTR complex (right eye). (A) Three-port 25G pars plana vitrectomy. (B) Core vitrectomy. (C) Dislocated IOL-CTR complex. (D) Removal of the CTR with forceps. (E) Extraction of the IOL through a small incision using Fukuoka forceps. (F) Gonioscopy confirming the widening of the angle. CTR: capsular tension ring; IOL: intraocular lens

To lower the IOP immediately, in the emergency outpatient clinic, an anterior chamber paracentesis was performed using a 20-G micro vitreoretinal (MVR) knife, and the IOL-CTR complex was pushed posteriorly. Intravenous mannitol was administered, and later that night, at 10:00 PM, surgery was performed to remove the IOL-CTR complex (Figures 4A-4F, Video 2). The surgery was conducted using a 25-G pars plana vitrectomy (PPV) system (Alcon Japan, Tokyo, Japan) (Figures 4A, 4B). The dislocated IOL-CTR complex was moved onto the iris surface (Figure 4C), and a 3 mm superior limbal incision was made for removal. The CTR was first removed (Figure 4D), followed by extraction of the IOL using Fukuoka forceps (HS-9868, Handaya, Tokyo, Japan) in combination with the IOL cartridge (Hoya) (Figure 4E). Gonioscopy confirmed that there was no PAS (Figure 4F), and the surgery was concluded. Postoperatively, levofloxacin and betamethasone eye drops were prescribed four times daily for about three weeks.

**Video 2:** Removal of the IOL-CTR complex (right eye). The video is being played at double speed. CTR: capsular tension ring; IOL: intraocular lens

**Figure 5:**
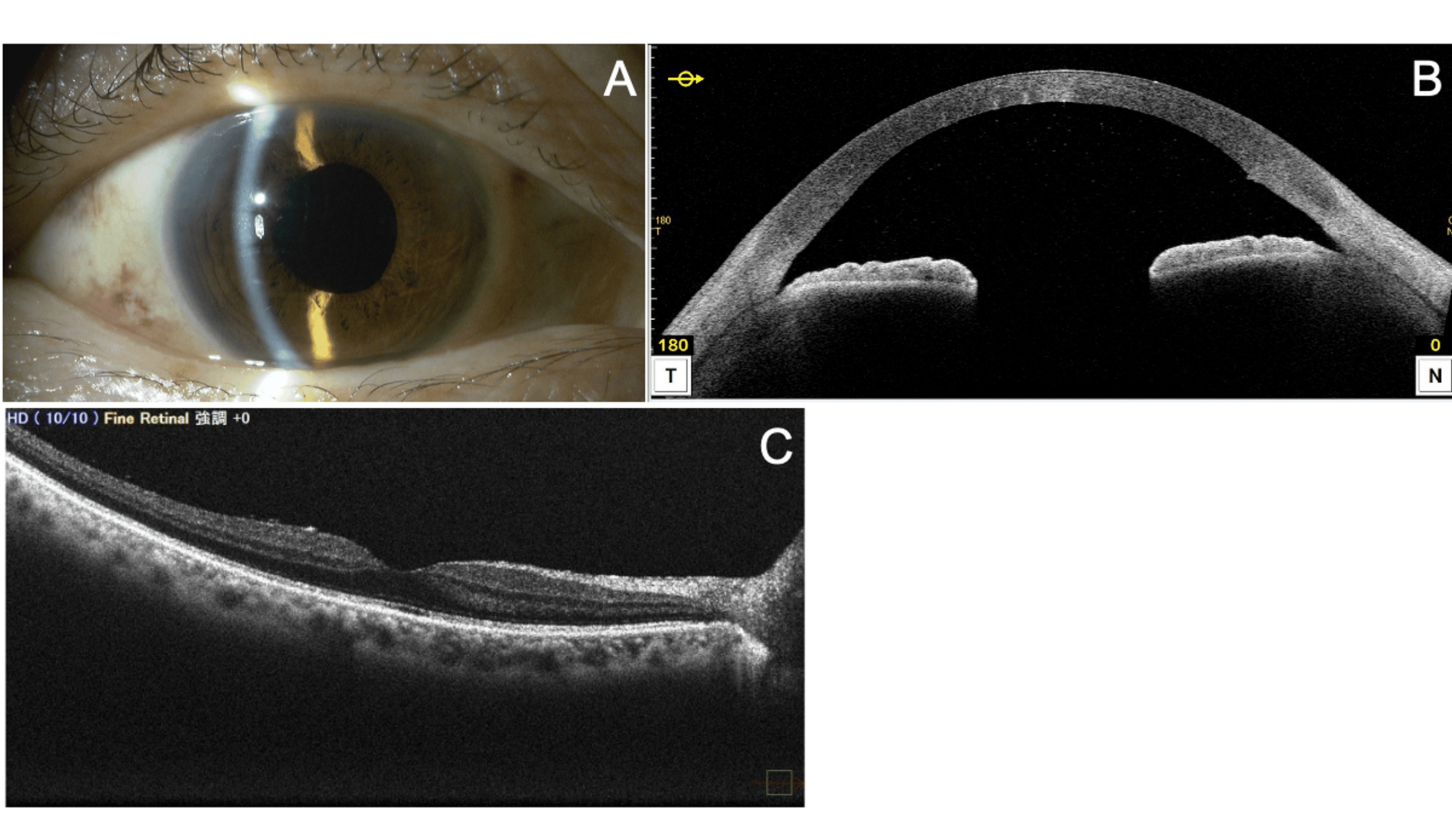
Postoperative findings five days after the removal of the IOL-CTR complex (right eye). (A) Slit-lamp examination shows a deep anterior chamber. The IOP is 4 mmHg. (B) AS-OCT (horizontal vertical scan) reveals a deepened anterior chamber, corneal edema, and ciliochoroidal detachment. (C) Fundus OCT (horizontal scan) shows no significant findings in the macula. AS-OCT: anterior-segment optical coherence tomography; CTR: capsular tension ring; IOL: intraocular lens; IOP: intraocular pressure

**Figure 6:**
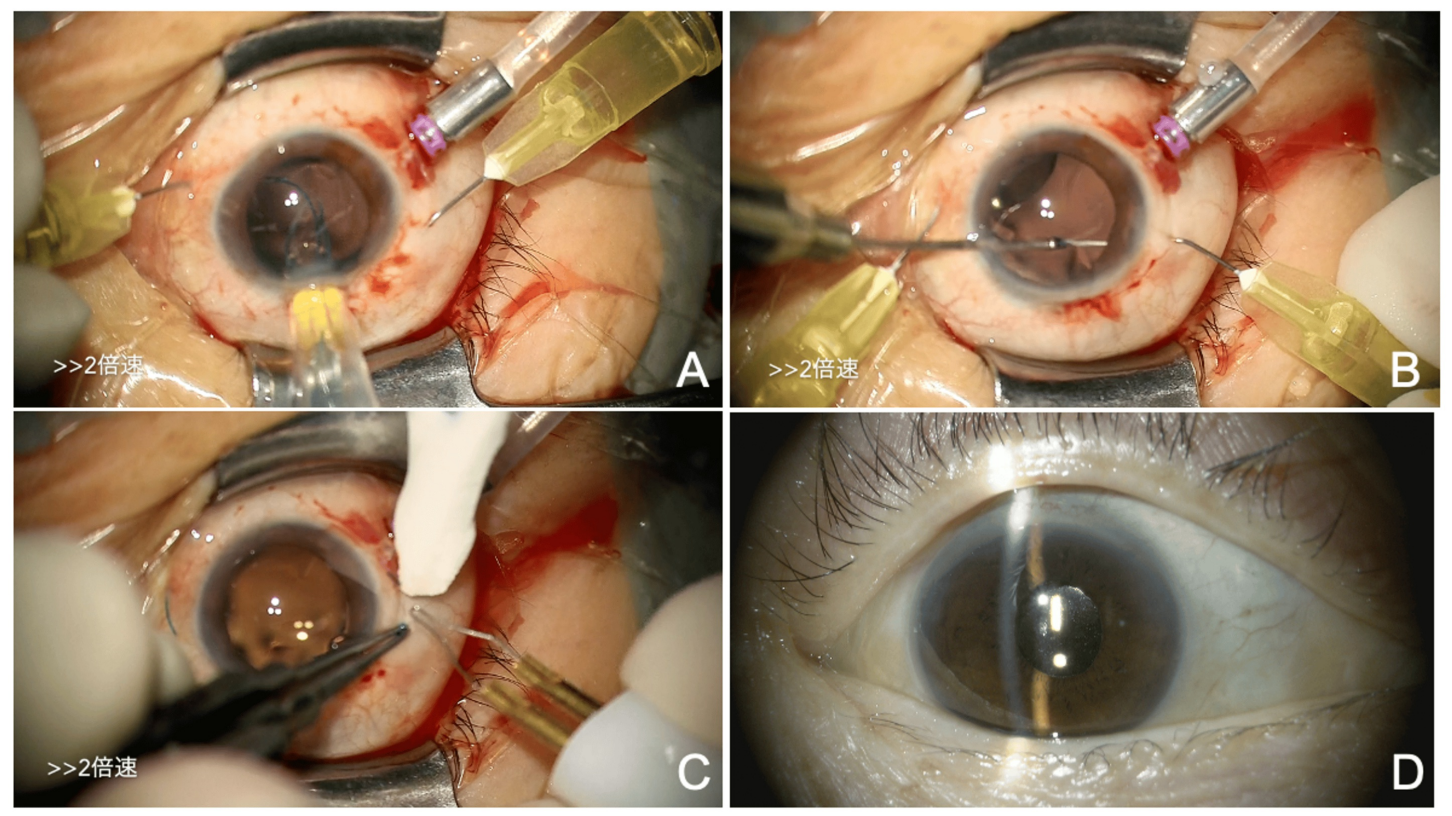
Intraoperative findings during scleral fixation of the IOL (A-C) and postoperative slit-lamp findings (D) (right eye). (A) IOL insertion through a superior small incision. (B) Externalization of the IOL haptics. (C) Flanged IOL fixation. (D) Two weeks after scleral fixation of the IOL, the IOL is well-centered and stable. IOL: intraocular lens

Five days post-surgery, BCVA in the right eye was (0.5 × S+7.0D), and IOP was 4 mmHg. The anterior chamber depth was deeper (Figure 5A). AS-OCT showed a deep anterior chamber and mild ciliochoroidal detachment (Figure 5B). Fundus OCT revealed no abnormalities in the macula (Figure 5C). Six weeks after IOL-CTR complex removal, scleral fixation of the IOL was performed (Figures 6A-6C, Video 3) using a 27-G PPV system (Alcon Japan). A +17.5D three-piece IOL (NX70S, Santen, Osaka, Japan) was inserted through a 3 mm superior limbal incision (Figure 6A). Using 30-G needles, the IOL haptics were passed through the sclera (Figure 6B), and the tips were thermally flanged to secure the IOL at the 4 and 10 o’clock positions (Figure 6C). Postoperatively, levofloxacin and betamethasone eye drops were prescribed four times daily for about three weeks. Two weeks postoperatively, BCVA in the right eye was (0.4 × S-1.0D = C-3.0D Ax 100°), and the IOP was 10 mmHg. The anterior chamber depth was deep, and the IOL was well-centered (Figure 6D).

**Video 3:** Intraoperative findings of IOL scleral fixation (right eye). The video is being played at double speed. IOL: intraocular lens

**Figure 7:**
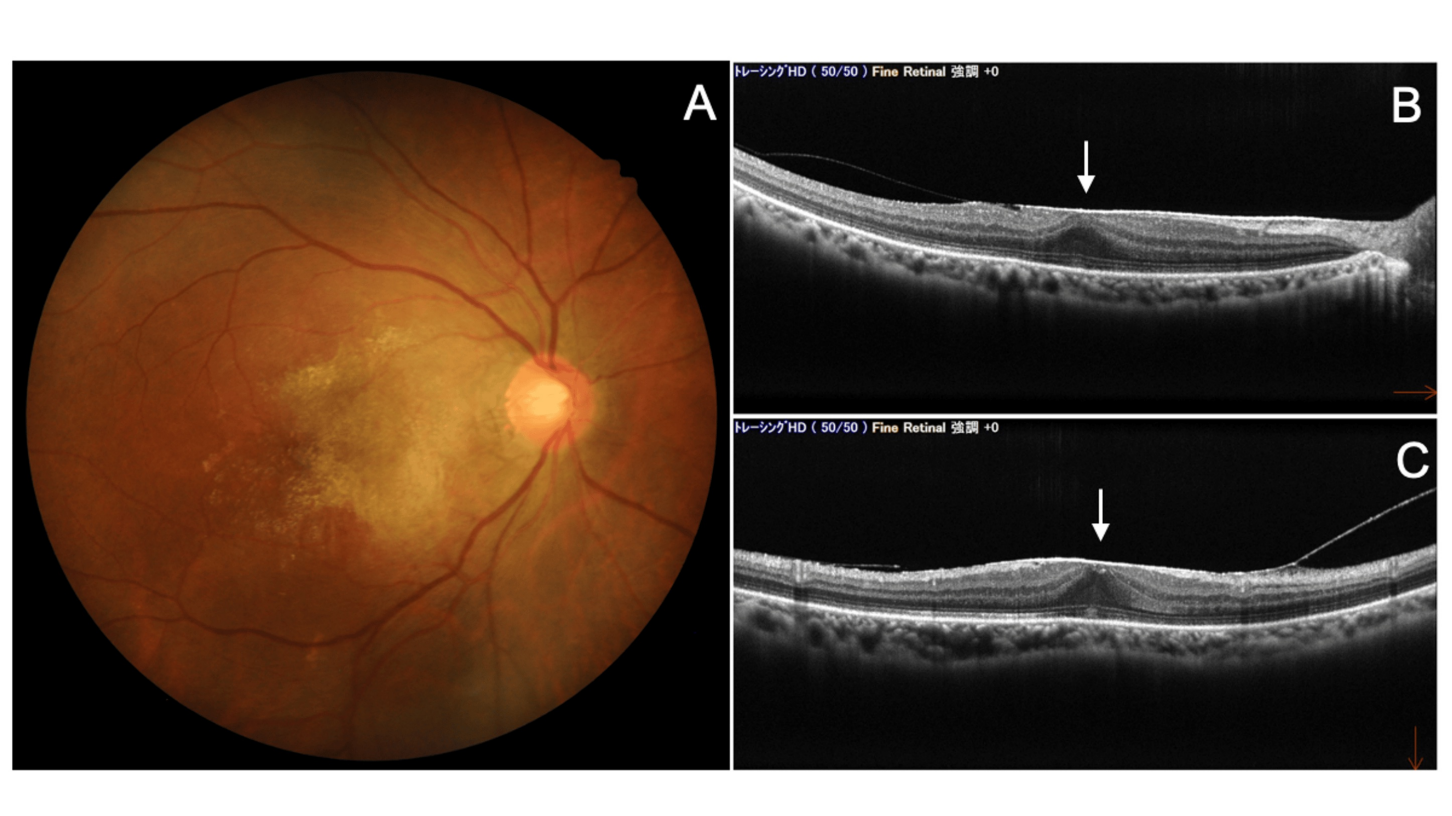
Fundus photo (A) and OCT findings (B) six weeks after scleral fixation of the IOL (right eye). (A) Fundus photo shows ERM formation. (B, C) Horizontal (B) and vertical (C) OCT scans of the macula reveal the loss of the foveal depression due to ERM traction (arrows). OCT: optical coherence tomography; IOL: intraocular lens; ERM: epiretinal membrane

**Figure 8:**
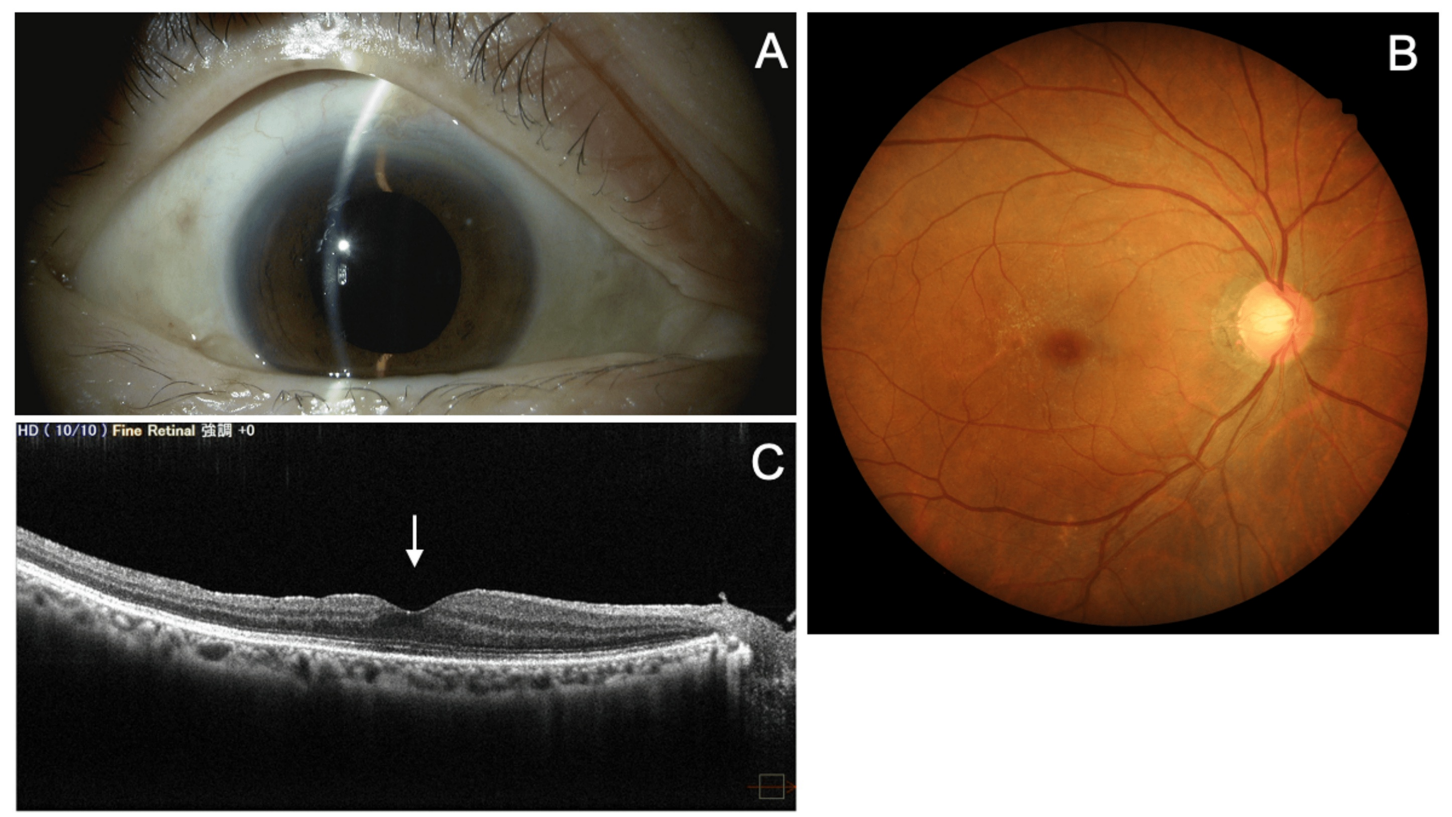
Final visit findings six months after IOL scleral fixation and four months after ERM surgery (right eye). (A) Slit-lamp examination shows that the anterior chamber depth is maintained. The IOP is 18 mmHg. (B) Fundus photo reveals a mild cellophane reflex at the macula. (C) OCT (horizontal scan) shows recovery of the foveal depression (arrow). OCT: optical coherence tomography; IOL: intraocular lens; ERM: epiretinal membrane; IOP: intraocular pressure

Six weeks after scleral fixation of the IOL, BCVA in the right eye was 1.0, and the IOP was 16 mmHg. A fundus examination revealed the formation of an epiretinal membrane (ERM) (Figures 7A-7C). Two weeks later, surgery was performed to remove the ERM using a 27-G PPV system (Alcon Japan). Postoperatively, levofloxacin and betamethasone eye drops were prescribed four times daily for about three weeks. At the final follow-up visit, eight months after the initial emergency visit, BCVA in the right eye was (1.2 × S+1.25D = C-2.5D Ax 95°), and the IOP was 18 mmHg without medication. The anterior chamber depth was deep, and the IOL was well-centered (Figure 8A). A mild cellophane reflex was observed in the macula (Figure 8B), but OCT showed that the traction from the ERM had resolved, and the foveal contour had recovered (Figure 8C). Corneal endothelial cell density (EM-3000) was 2,415 cells/mm², and central corneal thickness (EM-3000) was 554 µm.

## Discussion

We experienced a case of acute angle closure that occurred 28 months after cataract surgery with the use of a CTR for angle closure caused by zonular weakness of unknown etiology. The AS-OCT findings at the time of acute angle closure were characteristic, showing circumferential anterior displacement of the iris (Figures 3C, 3D). Previous reports have described AS-OCT findings [15] and ultrasound biomicroscopy findings [14] in cases of acute angle closure caused by a dislocated IOL-CTR complex. However, the images presented in those cases did not show the prominent iris compression seen in our case. In past cases, aqueous misdirection has been suggested as a cause of increased IOP [14,15]. In our case, as the anterior vitreous membrane remained posteriorly, and pushing the IOL-CTR complex posteriorly with an MVR knife during the emergency procedure resulted in a reduction of IOP before performing PPV, we infer that the mechanism of angle closure and IOP elevation in our case was more likely related to direct iris compression and pupillary block caused by the IOL-CTR complex rather than aqueous misdirection. Our case provides insights into the mechanism of IOP elevation due to a dislocated IOL-CTR complex.

There are various approaches to the removal of a dislocated IOL-CTR complex. In our case, we first removed the CTR, followed by the IOL using special forceps. When grasping the capsular bag along with the CTR, the capsular bag easily ruptured, allowing us to remove only the CTR. This method allows both the CTR and IOL to be extracted without cutting the devices, through an approximately 3 mm incision, resulting in minimal time under low IOP and being a minimally invasive approach. In this case, scleral fixation of the IOL was performed as a separate surgery. In acute glaucoma cases, there is often not enough time to prepare the IOL for emergency surgery. Additionally, postoperative inflammation or fundus complications may occur. Performing scleral fixation of the IOL as a separate surgery simplifies the procedure and is safer compared to combining it with the IOL-CTR complex removal. In this case, secondary treatment for postoperative ERM was also necessary. This case highlights the importance of comprehensive management in treating angle closure caused by a dislocated IOL-CTR complex.

## Conclusions

We experienced a case where a pupillary block caused by a dislocated IOL-CTR complex was clearly observed. In cases of secondary angle closure due to dislocated IOL-CTR complexes, proper diagnosis and comprehensive treatment management are crucial to achieving good IOP control and visual function.

## References

[1] Bayraktar S, Altan T, Küçüksümer Y, Yilmaz OF (2001). Capsular tension ring implantation after capsulorhexis in phacoemulsification of cataracts associated with pseudoexfoliation syndrome. Intraoperative complications and early postoperative findings. J Cataract Refract Surg.

[2] Chen J, Lan L, Tang Y, Lu Y, Jiang Y (2020). Placement of dual capsular tension rings for the combined management of traumatic cyclodialysis cleft and zonular dialysis. Eye Vis (Lond).

[3] Dai Q, Fu L, Liu XY, Pan WH (2021). Effective treatment for secondary angle-closure glaucoma caused by traumatic lens subluxation: phacoemulsification with capsular-tension-ring implantation combined with ophthalmic endoscope-controlled goniosynechialysis. Int J Ophthalmol.

[4] Lu Z, Wang L, Ying X, Tan L (2023). Bilateral angle closure glaucoma with retinitis pigmentosa in young patients: case series. BMC Ophthalmol.

[5] Pradhan C, Khadka S, Joshi P (2020). Angle closure glaucoma in retinitis pigmentosa. Case Rep Ophthalmol Med.

[6] Ma X, Li Z (2014). Capsular tension ring implantation after lens extraction for management of subluxated cataracts. Int J Clin Exp Pathol.

[7] Solmaz N, Oba T, Onder F (2023). Combined capsular tension ring and segment implantation in phacoemulsification surgery for the management of microspherophakia with secondary angle-closure glaucoma. Beyoglu Eye J.

[8] Salimi A, Fanous A, Watt H, Abu-Nada M, Wang A, Harasymowycz P (2021). Prevalence of zonulopathy in primary angle closure disease. Clin Exp Ophthalmol.

[9] Kubota T, Toguri I, Onizuka N, Matsuura T (2003). Phacoemulsification and intraocular lens implantation for angle closure glaucoma after the relief of pupillary block. Ophthalmologica.

[10] Lee BW, Lau FS, Wong EL, Lam D, Francis IC (2021). Lessons from management: perioperative phacoemulsification planning following resolution of acute angle closure. Cureus.

[11] Nakagawa S, Totsuka K, Okinaga K, Takamoto M, Ishii K (2024). Background factors determining the time to intraocular lens dislocation. Int Ophthalmol.

[12] Shingleton BJ, Neo YN, Cvintal V, Shaikh AM, Liberman P, O'Donoghue MW (2017). Outcome of phacoemulsification and intraocular lens implantion in eyes with pseudoexfoliation and weak zonules. Acta Ophthalmol.

[13] Werner L, Zaugg B, Neuhann T, Burrow M, Tetz M (2012). In-the-bag capsular tension ring and intraocular lens subluxation or dislocation: a series of 23 cases. Ophthalmology.

[14] Bochmann F, Stürmer J (2017). Chronic and intermittent angle closure caused by in-the-bag capsular tension ring and intraocular lens dislocation in patients with pseudoexfoliation syndrome. J Glaucoma.

[15] Goto K, Tomita R, Hiraiwa J, Kawabe M, Nishiguchi KM, Yuki K (2024). Secondary angle closure caused by anterior displacement of capsular tension ring and intraocular lens due to aqueous misdirection. Cureus.

